# An Engineering Approach to Biomedical Sciences: Advanced Testing Methods and Pharmacokinetic Modeling

**Published:** 2012-10-11

**Authors:** Gaetano Lamberti, Sara Cascone, Giuseppe Titomanlio

**Affiliations:** Dipartimento di Ingegneria Industriale, Università di Salerno, Salerno, Italy

**Keywords:** in-vitro, in-vivo, in-silico, pharmacokinetics, testing methods

## Abstract

In this paper, the philosophy of a research in pharmacology field, driven by an engineering approach, was described along with some case histories and examples. The improvement in the testing methods for pharmaceutical systems (in-vitro techniques), as well as the proposal and the testing of mathematical models to describe the pharmacokinetics (in-silico techniques) are reported with the aim of pointing out methodologies and tools able to reduce the need of expensive and ethical problematic in-vivo measurements.

## I. INTRODUCTION

The translational medicine is the process of turning appropriate biological discoveries into drugs and medical devices that can be used in the treatment of patients [1]. The basics of this new approach is that “the best results are achieved through the cooperative efforts of different disciplines, all aimed at the same objective” [1].

Within this frame, several researches were carried out in our group (Transport Phenomena and Processes, www.minerva.unisa.it) with the aim of bringing the engineering approach in the early stages of investigations in pharmacology and biomedical science [2].

Motivated by the guiding principles stated by FDA: “The basic principle in an in vivo bioavailability study is that no unnecessary human research should be done” [3], our research is focused on the development of in-vitro and in-silico tools for the investigation of the pharmacokinetics of drugs. Both the approaches were developed with the aim of limiting the use of unnecessary animal and human tests, replacing them with laboratory tests [4], [5].

With reference to the in-vitro tools, the starting point was the use of conventional tests, approved by USP (United States Pharmacopoeia). The most widespread technique, based on the Apparatus II (thermostated vessels stirred by paddles: size and shape of vessel and paddle being standardized), has some major drawbacks since it cannot simulate the real physiology of gastrointestinal tract (GI): since the motion of fluids (the fluid-dynamic) and the biochemical history (mainly the pH) are different in the test apparatus and in the real GI tract, the absorption across the intestinal wall is not simulated at all in the test apparatus. Therefore, there is room for significant improvements of the commonly used apparatuses and techniques [6].

The in-silico approaches require the definition of a proper pharmacokinetics model which, after a simple and limited validation session (during which some in-vivo tests have to be considered), should act as a predictive tool. This means that the model, properly calibrated, could predict the hematic level of the drug after its administration to the human body, even if the administration route was changed or the drug delivery system was modified with respect to the administration route/the drug delivery system adopted during the in-vivo tests.

Aim of this work is to describe the approaches and the main results of our group within this two fields.

## II. METHODOLOGY

### A. In-vitro methodology

To test enteric dosage forms, the USP recommend a two stage procedure [8]. During the first stage (acid stage), the solid dosage form should be inserted into a vessel containing 750 mL of HCl 0.1 M (pH 1.0); then, after two hours, the pH has to be raised to 6.8 by adding 250 mL of 0.2 M Sodium Phosphate Tribasic (buffer stage).

To allow a full control of the pH into the dissolution vessel, 500 mL of a solution buffer with initial pH of 4.8 was inserted into the vessel (to mimic the real pH in the stomach just after the end of a meal), and a given pH history was then reproduced by means of an apparatus designed and built to the purpose. The apparatus was able to dose acid (HCl 2 M) or base (NaOH 2 M) solutions into the vessel. After two hours, the sudden pH raise which has to mimic the passage toward the intestine was realized by adding a given amount of NaOH solution, and then allowing the control system to guarantee the realization of the set-up value of 6.8.

To control the pH in the dissolution medium into one of the Apparatus 2 vessel, two peristaltic pumps were used to dose the acid and the base solutions. The pH is measured by using a glass pH – probe. A data acquisition board was used to measure and collect the sensor signals. A dedicated software was developed to provide the control of the pH and the temperature evolution in the *ad-hoc* realized device [9].

The tests were carried out on commercial tablets (extended release - enteric coated, Diclofenac EG, Eurogenerici, Milan, Italy). Each tablet contains 100 mg of the active molecule (Diclofenac Sodium, DS). The tablets were enteric coated with a polymer insoluble at low pH, to avoid the release into the stomach, the other excipients being selected to produce a slow release (extended release) at higher pH, once the tablet reaches the intestine.

### B. In-silico methodology

**Fig. 1:**
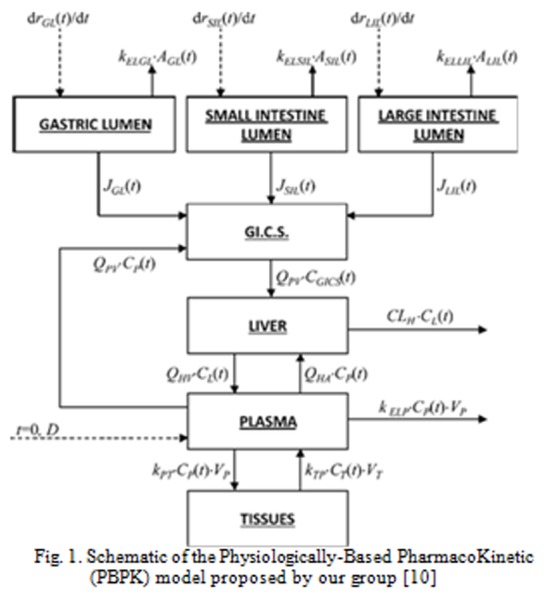
Schematic of the Physiologically-Based PharmacoKinetic (PBPK) model proposed by our group [10]

The PBPK model developed by our group was based on a simple representation of the body, reported in Fig. 1[10]. Each of the blocks represents an organ/a tissue/a fluid of the body or a group of them: the gastrointestinal tract is split into the stomach, the small intestine and the large intestine; the gi.c.s block represents the gastrointestinal circulatory system; the liver block represents the hepatic compartment; the plasma block represents the blood and the largely perfused tissues and organs; the tissues compartment represents the scarcely perfused tissues and organs. The continuous arrows represent the mass flow rates between the compartments. The dashed arrows represent the drug inlets after administration by intravenous or oral route: in the case of intravenous injection (i. i.), the only dashed arrow which needs to be considered is that entering the plasma compartment. Otherwise, in the case of oral assumption (o. a.), the dashed arrows which need to be considered are those entering the gastric lumen (from ingestion to gastric emptying), the small intestine lumen (from gastric emptying to small intestine emptying) and the large intestine lumen (from small intestine emptying to large intestine emptying), depending on where the drug is released. The assumption made in doing mass balances are that the blocks can be assimilated to continuous stirred reactors and that the mechanism of drug transport across biological membranes (e.g. the gastrointestinal walls and the blood vessels walls) can be assimilated to passive diffusion.

The PBPK model consists of the mass balance equations on the compartments coupled with their initial conditions. Obviously, several parameters need to be known in order to solve the system of equations. Namely, the model consists in 7 ODEs (Ordinary Differential Equations) and it needs the knowledge of 22 parameters, some of them could, in principle, be obtained by fitting some in-vivo data (taken by literature). The solution of model equations has to be achieved numerically, and the equations have been implemented using commercial software (Mathcad, Matlab^®^).

## III. RESULTS AND DISCUSSION

### A. In-vitro results

One of the most important environmental factor affecting the drug release from solid dosage forms, administered orally, is the pH. In fact, the drug delivery system experiments several pH conditions during its traveling along the GI tract: a low pH in the stomach (about 4–5), followed by an almost neutral pH in the small intestine, followed by a further rise in pH ( about 7) in the large intestine. Since the adsorption in the stomach is not very effective, to avoid drug degradation the ideal drug delivery system should not release any drug in the stomach (at low pH), then it should release its drug content in the small intestine where the absorption is the largest (the best would be if the release takes place at a constant rate, to replace exactly the drug metabolized: the so-called zero-order drug delivery system). Only in special cases, the drug release has to be targeted to the colon (e.g. to treat localized diseases). Therefore, the design of drug delivery systems has been addressed to the production of the so-called “enteric” systems, i.e. tablets which does not release any drug at the low pH values expected in the stomach, delivering their drug content once the pH raises to neutral values.

**Fig. 2:**
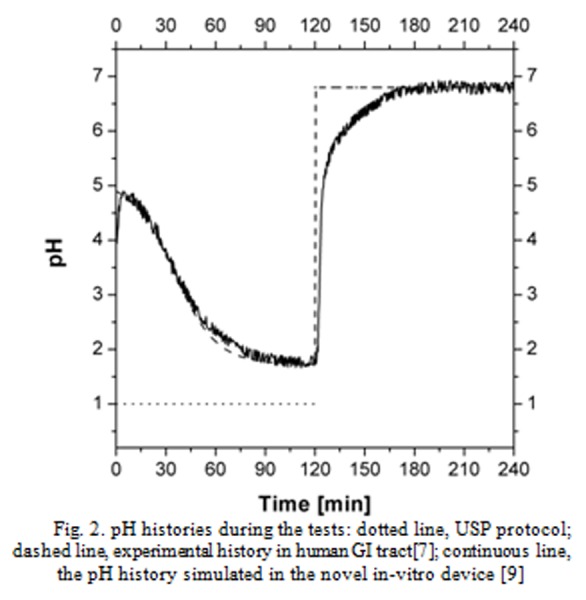
pH histories during the tests: dotted line, USP protocol; dashed line, experimental history in human GI tract [7]; continuous line, the pH history simulated in the novel in-vitro device [9]

To test the effectiveness of such drug delivery systems, a two stages protocol (briefly summarized in the Methodology section) is reported in the USP. In Fig. 2 the related pH history is reported as a dotted line: the pH was kept on pH 1.0 for the first two hours, to mimic the passage in the stomach; then it was raised to 6.8 to mimic the passage in the small intestine. In the real human physiology, the pH in the stomach is not equal to 1.0. In fed state, it is roughly 4.8–5.0 and it starts to decrease after a meal. Then, once the bolus passes the pylorus, the pH increases toward neutral values. Therefore, a more realistic pH history is drawn in Fig. 2 as a dashed line, taken from literature, measured in-vivo after a light meal [7]. It is evident that the decrease in pH is not very fast, and during the two hours of staying in the stomach, the pH barely reach the value of pH 2.0. The set-up described in the Methodology section allowed us to simulate the realistic pH history in the vessel of Apparatus II: the “real” pH history is reported as a black continuous line.

**Fig. 3:**
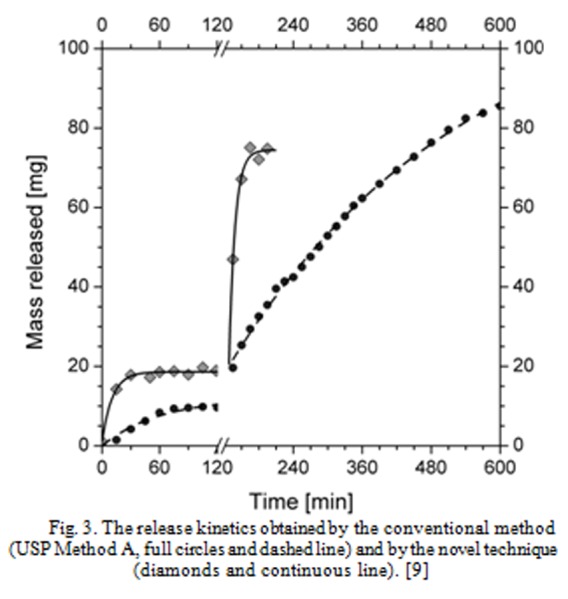
The release kinetics obtained by the conventional method (USP Method A, full circles and dashed line) and by the novel technique (diamonds and continuous line). [9]

Therefore, we could analyze the release kinetics in the two environments, with different pH evolutions: 1) using the conventional enteric test (the pH history being given by the dotted line in Fig. 2); and 2) using the novel test protocol depicted above (the pH history being given by the continuous line in Fig. 2). The results of these two tests are reported in Fig. 3. The full circles are the experimental result of the release kinetics observed by the traditional (USP Method A) protocol, the diamonds are the experimental result of the release kinetic data observed by the novel test protocol. It is evident that:

1. in the conventional USP test, during the acid stage, a very little amount of drug is released (less than 10 mg, the total content being 100 mg). The release in the buffer stage is well controlled (after 8 hours, less than 90 mg were released).
2. in the novel test (which mimics the real physiology better than the conventional test), during the acid stage, the drug release is high (20 mg) and the remaining drug is released during the buffer stage very quickly (less than one hour to release about 80 mg).

Summarizing, the release in the real stomach would be more than the twice the release in the conventional in-vitro test (USP); and the release in the real intestine would occur in a very short time (in the conventional in-vitro test it takes more than 8 hours). This could be due to the fact that the coating technique is insufficient to guarantee the enteric behavior, as the excipients have to be resistant to unexpected high pH levels. Possible solutions could consist in the use of different preparation method/materials (e.g. microencapsulation / polyacrilic polymers), or in the indication of a proper administration therapy (e.g. the tablet should be dosed one hour after the end of the meal). It should be noted that the administration of these tablets, fully compliant with the USP tests, could be ineffective; and that a deeper understanding of what happens once the pill has been swallowed derived from the novel “engineering” approach to the problem” [9].

### B. In-silico results

Usually, pharmacokinetic modeling is aimed to establish correlations between the in-vitro release data such as the release reported in Fig. 3 with in-vivo hematic drug concentration (or different measurements of the drug in the bodies of living beings). If the model consists in purely mathematical equations, without any physical meanings, they are called IVIVC (In-Vitro In-Vivo Correlations) and they are classified on the basis of their ability to correlate the two profiles. Regulatory boards (FDA [12], USP [13]) accept only the higher-level correlations (the so-called level A IVIVC) for Scale-Up and Post-Approval Changes (SUPAC) which “can be justified without the need for additional human studies”.

Of course, a PK model able to correlate the in vitro dissolution profile and the in vivo plasma evolution, will play the same role of a Level A IVIVC, but being more physically based, it would be of major interest for researchers. This was the motivation of the design and the coding of the model whose main parts are reported in the scheme of Fig. 1.

**Fig. 4:**
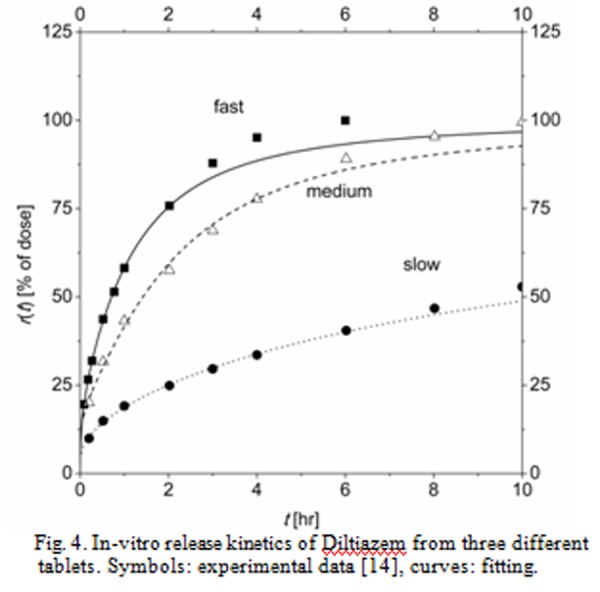
In-vitro release kinetics of Diltiazem from three different tablets. Symbols: experimental data [14], curves: fitting.

**Fig. 5:**
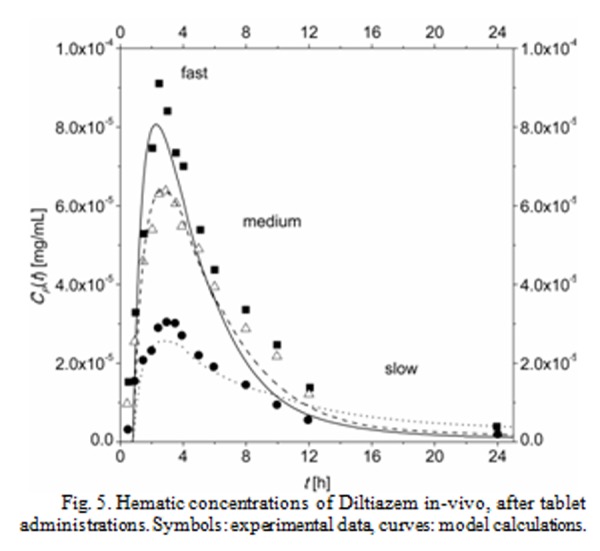
Hematic concentrations of Diltiazem in-vivo, after tablet administrations. Symbols: experimental data, curves: model calculations.

For example, Fig. 4 reports the in-vitro release kinetic data and Fig. 5 reports the related in-vivo hematic concentration of Diltiazem administered orally to healthy volunteers. The symbols in both the graphs are experimental data, taken from ref. [14]; Diltiazem is a calcium channel blockers, used among other therapies in the treatment of hypertension. In Fig. 4 three series of date were displayed, representing the in-vitro release kinetics of three different tablets, labeled ‘fast’, ‘medium’ and ‘slow’ because of the decreasing release rate. Similarly, in Fig. 5 three series of data are displayed, representing the in-vivo hematic concentration evolution due to the administration of the three different tablets before tested in-vitro (the main difference between the three evolutions being the maximum drug hematic concentration and the Area Under the Curve, which is a measure of bioavailability).

The process of pharmacokinetic modeling required some steps:

1. First of all, the in-vitro release kinetics was fitted by suitable mathematical expression. This step was required because the in-vitro data are inputs of the physiologically base pharmacokinetics (PBPK) model, and scattered experimental data do not play well in numerical code, mainly because of sudden changes in derivative. Results of this fitting session are the curves in Fig. 4.
2. Then, the PBPK model was used to simulate the hematic concentration due to the administration of the ‘medium’ rate tablet. During this step, the model parameters were adjusted to enable the model predictions to describe the real in-vivo drug concentration evolution. Result of this fitting session is the dashed curve in Fig. 5 which ***describe*** the in-vivo behavior. It is worth to note that this step does require the analysis of some results of in-vivo experiments.
3. At this point, the model is ***predictive***, i.e. it should be able to predict the drug hematic concentration after the administration of a tablet with release kinetics different than the ‘medium’ rate one. Actually, the curves in Fig. 5 for ‘fast’ and ‘slow’ rate administration (respectively, continuous and dotted curves) have been calculated with the model without any further optimization work.

It is worth to note that the model predicts the real maximum drug concentrations and AUCs for ‘fast’ and ‘slow’ rate tablets accurately enough. As matter of facts, the predictions offer the same level of accuracies of a Level A IVIVC, therefore the PBPK model, in principle, could be used in SUPAC process accordingly with regulatory boards.

The two series of data (‘fast’ and ‘slow’ rate) are thus unnecessary: the availability of the tool constituted by the PBPK model could avoid a large number of in-vivo experiments, which are ethically problematic and resource asking.

## IV. CONCLUSIONS

The translational research could be a powerful tool for researchers over all the world to guide the process “from bench to bedside”, i.e. the translation of non-human research finding, from the in-vitro and from in-silico studies, into human therapies. The translational medicine found its roots into interdisciplinarity, since it is based on the creation of something new by thinking across the boundaries between different scientific disciplines. Within this contest, the engineering approach is of tremendous utility in pharmacology and in biomedical science.

In this work, two case histories are reported.

In the first one, an improvement in the in-silico testing for solid dosage forms showed how a tablet, designed to give a well-defined release kinetics – and proved able to work efficiently by traditional test methods – was found very ineffective once tested in the novel test method, which better reproduces real physiological conditions. By this study, the need for proper drugs administration therapies was pointed out.

In the second case history, a simple and effective physiologically based pharmacokinetic model was proposed and tested; it was proved to describe nicely the hematic levels of several drugs, even for different administration routes. The model could be the basis for more detailed pharmacokinetic studies (varying also inter-individual parameters such as age, gender and weight) toward the development of the personalized therapies.
